# Equity of Multidisciplinary Hip-Fracture Care in Patients With Dementia: Time-to-Theatre and Discharge Outcomes: A Retrospective Cohort Study

**DOI:** 10.7759/cureus.96606

**Published:** 2025-11-11

**Authors:** Mustafa Mouhsen, Abdelaziz Elsalmawy, Khadijah Boujettif, Mohamed Elgamal, Layth Qazzaz, Abuamar Zaidan

**Affiliations:** 1 Trauma and Orthopaedics, Southend University Hospital (Mid and South Essex NHS Foundation Trust), Southend-on-Sea, GBR; 2 Orthogeriatrics, Southend University Hospital (Mid and South Essex NHS Foundation Trust), Southend-on-Sea, GBR; 3 Geriatrics, Southend University Hospital (Mid and South Essex NHS Foundation Trust), Southend-on-Sea, GBR

**Keywords:** cognitive impairment and dementia, equitable surgical care, neck of femur fractures, orthogeriatric care, time to surgery

## Abstract

Background

Cognitive impairment is common among hip fracture patients and may influence care and outcomes. We evaluated whether patients with dementia or delirium receive equitable multidisciplinary hip-fracture care compared with cognitively intact patients, focusing on surgical timing and discharge outcomes.

Methods

From 775 admissions, we retrospectively reviewed a random sample of 382 cases (January 2024-June 2025) at Southend University Hospital, stratifying into impaired (dementia, suspected cognitive decline, or delirium on admission; n=191) and non-impaired (normal cognition on Abbreviated Mental Test Score [AMTS]/4AT; n=191) groups. Using the National Hip Fracture Database, we collected data on time-to-surgery, orthogeriatric review, preoperative 4AT, physiotherapy and mobilisation, pain management, communication support, carer involvement, do not attempt cardiopulmonary resuscitation (DNACPR) documentation, discharge destinations, and in-hospital mortality. Outcomes were compared using chi-square for categorical data and t-test or Mann-Whitney for continuous data, with p<0.05 significant.

Results

Impaired patients experienced longer delays to theatre (median 35.3 h [IQR 23.0-48.7] vs. 29.8 h [20.6-43.8]; mean 44.9±36.0 vs. 36.5±32.3; p=0.029). Time to orthogeriatric review was similar (median ~21 h in both). Not all impaired patients had 4AT ≥4, but the median was higher in the impaired group (4 vs. 0). Impaired patients were less likely to return home (52.7% vs. 84.4%, p<0.001), more often discharged to residential care (35.3% vs. 7.5%), and had higher in-hospital mortality (8.7% vs. 2.7%, p=0.02). Physiotherapy assessment rates were high (>97%), but early mobilisation was lower in impaired patients (81.6% vs. 89.9%, p=0.04), with agitation frequently noted as a barrier. Analgesic prescribing was similar, but the qualitative review suggested undertreatment in impaired patients.

Conclusions

Patients with cognitive impairment experienced slower access to theatre, reduced early mobilisation, a lower likelihood of discharge home, and higher in-hospital mortality. Dementia-informed adjustments, such as fast-track surgery, validated pain tools, delirium-prevention bundles, carer engagement, and strengthened rehabilitation, are warranted to deliver equitable hip-fracture care.

## Introduction

Hip fracture represents a global health crisis with substantial morbidity and mortality; one-year mortality commonly approaches 20-30% even with surgical management [1]. Timely surgery and orthogeriatric co-management are central to current care pathways. Delays of >24-48 have been associated with higher short-term mortality and worse functional recovery [2-4], whereas early surgery facilitates earlier mobilisation and return to independent living [5]. Consequently, national standards emphasise theatre within 36 h, prompt geriatric assessment, and day-one mobilisation [6,7].

Cognitive impairment, encompassing pre-existing dementia and acute delirium, is prevalent in hip-fracture populations and significantly complicates peri-operative care [8]. Dementia is associated with increased short-term and one-year mortality after hip fracture [1] and a greater need for institutional care after discharge [9-11]. Despite this risk, most patients, including those with advanced dementia, are managed operatively because repair improves comfort and survival compared with nonoperative care when appropriate [12]. The challenge for modern orthogeriatric services is to ensure that the benefits of prompt, standardised care are delivered equitably across the entire patient population, including these vulnerable individuals. Early identification of delirium using validated tools such as the 4AT is recommended on admission to guide targeted delirium prevention and management [13].

Knowledge gap

Modern orthogeriatric pathways are designed to deliver equitable, standardised care; however, it remains uncertain whether patients with cognitive impairment experience systematic delays or differential peri-operative processes within these pathways. Specifically, potential disparities may arise around consent processes, prioritisation for theatre, analgesia delivery, and mobilisation.

Objectives

We compared time-to-theatre and discharge outcomes between patients with and without cognitive impairment within a contemporary orthogeriatric service. Secondary objectives were to compare the timing of geriatric assessment, early mobilisation, analgesia administration, communication and capacity documentation, and advance-care planning. We hypothesised that patients with dementia or delirium would experience longer surgical waits, less successful early mobilisation, and lower rates of discharge home.

## Materials and methods

Study design and setting

Retrospective cohort study at Southend University Hospital, UK. All consecutive patients aged ≥60 years admitted with fragility hip fracture between 1 January 2024 and 30 June 2025 were screened (N=775).

Participants (patient recruitment)

From the sampling frame, a random sample of 382 cases was selected a priori and split equally by cognitive status into impaired and non-impaired groups (n=191 per group). Equal allocation (191/191) was used to maximise precision of between-group comparisons at a fixed sample size; prevalence estimation was not the aim. Inclusion criteria: age ≥60, radiologically confirmed fragility hip fracture, and operative pathway intended. Exclusion criteria: periprosthetic or pathological fractures due to malignancy, nonoperative palliative pathway at admission, or missing core timestamps precluding analysis of the primary process measure.

Cognitive stratification (exposure definition)

“Impaired cognition” included documented dementia, active investigation for cognitive decline, or delirium on admission. Delirium screening used the 4AT; a score ≥4 indicated probable delirium [13]. Not all patients with known dementia scored ≥4. The comparator group had preserved cognition on admission.

Variables (outcomes and covariates)

Primary process outcome: admission-to-surgery time (hours). Other care-process measures: admission-to-orthogeriatric review; analgesia prescription and administration in the first 72 h; physiotherapy assessment and mobilisation by postoperative day (POD) 1-2; documentation of communication needs, carer involvement, capacity/consent, and DNACPR. Clinical outcomes: discharge destination (home vs. institutional care) and in-hospital mortality. These processes reflect nationally recommended best-practice elements (orthogeriatric co-management, early mobilisation) associated with improved outcomes [6,10,14].

Data sources and measurement

Data were abstracted from the National Hip Fracture Database (NHFD), theatre systems, and therapy notes. Time stamps used system-generated times for admission, geriatric review, and anaesthetic start. Analgesia data captured both prescriptions and administrations. Mobilisation was coded as “out of bed” by POD1-2 based on physiotherapy notes.

Bias

To mitigate selection bias, we used random sampling from NHFD. Information bias was reduced by double-checking a 10% sample against source records with >95% agreement for key timestamps. Misclassification of delirium was limited by using the 4AT at admission [13].

Study size (sample size calculation/justification)

Because this was a retrospective study with a fixed accrual window, we drew a priori a random sample of 382 cases (191 per group) to balance feasibility and precision. For two independent proportions, the required per-group size can be approximated by the Fleiss formula [15]:

$n  =  \frac{\left[z_{1&minus;\alpha/2}\sqrt{2\bar{p}(1&minus;\bar{p})} + z_{1&minus;\beta}\sqrt{p_1(1&minus;p_1)+p_2(1&minus;p_2)}\right]^2}{(p_1&minus;p_2)^2}$

with two-sided α=0.05\alpha=0.05α=0.05 and power 1−β=0.801-\beta=0.801−β=0.80. Using n=191n=191n=191 per group, the study has ~80% power to detect an absolute difference of ≈13% when the average event rate is ~0.7 (e.g., discharge-home proportions) and ≈14% when the average event rate is ~0.5. Precision for key estimates was acceptable: the 95% CI half-widths were ~7.1% for discharge home in the impaired group (52.7%), 5.1% for non-impaired (84.4%), 4.0% for in-hospital mortality in the impaired group (8.7%), and 2.3% for non-impaired (2.7%). In this service-evaluation context, the chosen sample size provides clinically useful precision for group comparisons and effect-size estimation.

Quantitative variables

Continuous variables were summarised as mean ± SD or median (IQR) after distribution checks; categorical variables as n (%). Mobilisation was treated as binary by POD threshold; analgesia administrations were counted over fixed windows.

Statistical analysis

Between-group comparisons used t-tests or Mann-Whitney U for continuous variables and χ² or Fisher’s exact tests for categorical variables. Two-sided α=0.05; 95% CIs are reported where informative. Analyses were descriptive and unadjusted; findings are interpreted alongside existing evidence [1-5,9,10,14,16]. IBM Corp. Released 20020. IBM SPSS Statistics for Windows, Version 29.0. Armonk, NY: IBM Corp was used.

Ethics

This retrospective analysis of routine care was registered locally as a service evaluation; identifiable data were not exported. Formal research ethics board review and individual consent were not required under local policy. Local approval was obtained.

## Results

Cohort and baseline characteristics

Of 775 eligible admissions, 382 randomly sampled cases formed the analytic cohort (n=191 impaired; n=191 non-impaired). Groups were similar in age (overall 82.6 ± 8.9 years) and sex (~70% female). By design, all impaired patients had dementia and/or delirium features; comparators had preserved cognition.

Time to surgery and geriatric review

**Figure 1:**
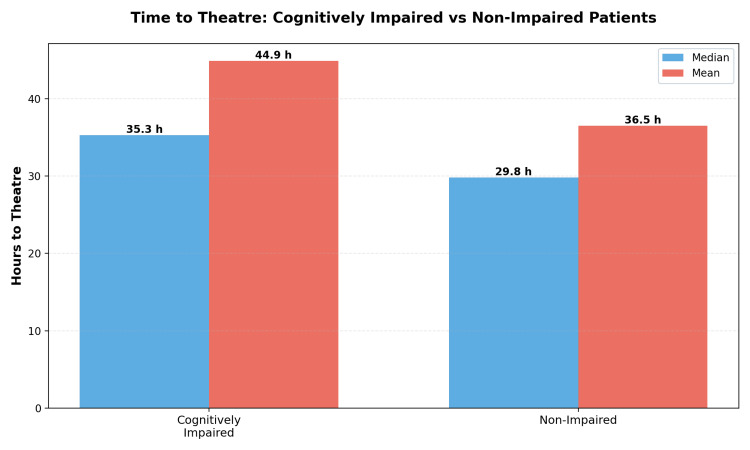
Comparison of time to theatre between cognitively impaired and non-impaired patients Bar chart comparing hours from hospital admission to anaesthetic start for patients ≥60 years with fragility hip fracture at Southend University Hospital (retrospective cohort, Jan 2024–Jun 2025). Data are shown for cognitively impaired (dementia and/or delirium) and non-impaired groups using median (blue) and mean (red) values; annotated labels give exact estimates (impaired: median 35.3 h, mean 44.9 h; non-impaired: median 29.8 h, mean 36.5 h). The analytic subset includes cases with complete timestamps (impaired n=152, non-impaired n=160). Between-group comparisons: Mann–Whitney U for medians and two-sample t-test for means (p=0.029 for mean difference; CIs not shown). Interpretation: cognitively impaired patients experienced a longer time to theatre than non-impaired peers within the same orthogeriatric pathway. h: hours

Among 312 patients with complete timestamps (152 impaired; 160 non-impaired), excluding 70 cases primarily due to missing admission or surgical start times, median time-to-theatre was 35.3 h (IQR 23.0-48.7) vs. 29.8 h (20.6-43.8) in the non-impaired group; means were 44.9 ± 36.0 vs. 36.5 ± 32.3 (p=0.029). Nearly half of impaired patients breached the ≤36 h standard [6] (Figure 1). Delays >48 h, previously linked to higher mortality, were more frequent in the impaired group [2-4]. Time to orthogeriatric assessment was similar (median ~21-22 h), indicating equitable geriatric access [14].

Analgesia processes

Baseline and PRN analgesia prescriptions were similar, but administration of PRN opioids in the first 24-48 h was lower in impaired patients compared to non-impaired patients, consistent with prior reports of undertreatment after hip fracture in dementia [17-19].

Physiotherapy and early mobilisation

Physiotherapy assessment occurred in ~97-100% across groups. By POD2, out-of-bed mobilisation was achieved less often in impaired patients (81.6% vs. 89.9%, p=0.04). Early mobilisation is associated with improved 30-day survival and recovery of walking across cognitive strata [10]. Agitation/delirium was a common barrier, supporting delirium-prevention bundles [10,20].

Discharge destination and in-hospital mortality

**Figure 2:**
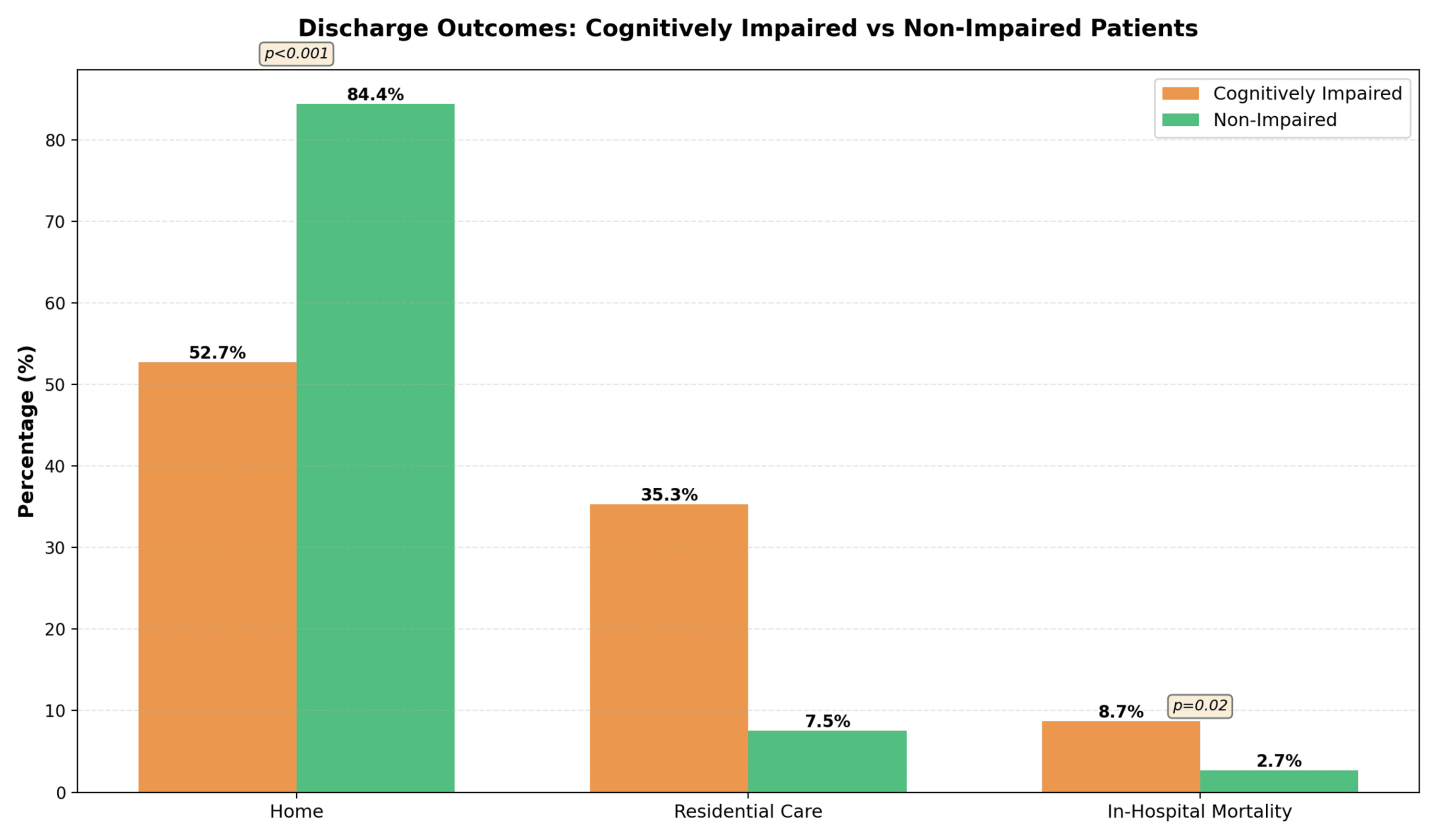
Comprehensive comparison of hip fracture care outcomes Clustered bar chart showing discharge outcomes for patients ≥60 years with fragility hip fracture treated at Southend University Hospital (Jan 2024–Jun 2025). Groups: cognitively impaired (dementia and/or delirium; n=191) and non-impaired (n=191). Bars display percentages for (i) Home discharge, (ii) Residential care (new or ongoing placement/step-down bed), and (iii) In-hospital mortality. Point estimates annotated on bars: Home 52.7% vs. 84.4%; Residential care 35.3% vs. 7.5%; In-hospital mortality 8.7% vs. 2.7%. Statistical methods: between-group comparisons by χ² test (overall p < 0.001 for discharge destination; p = 0.02 for mortality); 95% CIs not shown. Interpretation: relative to non-impaired patients, those with cognitive impairment were less likely to be discharged home, more likely to require residential care, and had higher in-hospital mortality.

Discharge home occurred in 52.7% of impaired vs. 84.4% of non-impaired patients (p<0.001); institutional placements were substantially higher in the impaired group, echoing registry findings [9,10]. In-hospital mortality was higher among impaired patients (8.7% vs. 2.7%, p=0.02) (Figure 2), aligning with meta-analytic evidence of increased early mortality in dementia [1].

## Discussion

Principal findings

In a contemporary orthogeriatric pathway, patients with cognitive impairment (dementia and/or delirium) experienced longer waits for surgery, were less likely to mobilise by POD2, were discharged home less often, and had higher in-hospital mortality than cognitively intact peers, despite similar time to orthogeriatric assessment. These inequities cluster around modifiable processes (consent/logistics, analgesia delivery, and early mobilisation), suggesting opportunities for targeted system redesign.

Comparison with existing literature

Our time-to-theatre difference (median 35.3 h vs. 29.8 h; mean 44.9 vs. 36.5 h) is consistent with reports that cognitively impaired patients face longer perioperative pathways, often related to consent and preoperative optimisation rather than medical contraindication per se. The observed proportion breaching ≤36 h aligns with studies linking delays >24-48 h to increased early mortality and complications [2-4]. While national standards codify ≤36 h access [6,7], our data indicate that dementia/delirium remains a risk factor for delay within otherwise standardised services.

Undertreatment of pain in dementia is well described; multiple cohorts show lower opioid administration despite comparable prescribing, with associations to worse agitation, delirium persistence, and impaired rehabilitation [17-19]. Our cohort reproduced this pattern: prescriptions were similar, but PRN opioid administration in the first 24-48 h was lower in the impaired group. This gap is likely due to multifactorial assessment challenges in non-verbal patients, safety concerns about oversedation/falls, and staff hesitancy without structured pain tools (e.g., PAINAD).

Mobilisation by POD2 was less frequent in impaired patients (81.6% vs. 89.9%), paralleling evidence that delirium and untreated pain impair participation in therapy sessions. Early mobilisation is independently associated with improved 30-day survival and recovery of walking across cognitive strata [10]; thus, the mobilisation gap represents a credible mediator of downstream outcomes. The markedly lower rate of discharge home and greater institutionalisation among cognitively impaired patients echoes registry data and meta-analyses [9,10], which attribute part of the effect to pre-morbid frailty and social support, but also to in-hospital processes (analgesia, mobilisation, and delirium management). Our higher in-hospital mortality in the impaired group (8.7% vs. 2.7%) is concordant with pooled estimates showing increased short-term and one-year mortality in dementia after hip fracture [1].

Potential mechanisms and pathways of effect

Consent and theatre logistics: capacity assessments, best-interest meetings, and next-of-kin/surrogate coordination introduce additional steps that can push cases beyond the 36-hour window if not pre-planned.

Anaesthetic/ward workflow: patients with agitation may be deprioritised for first-start lists or require additional pre-op optimisation, extending queues.

Pain assessment/treatment: without observational scales and scheduled non-opioid regimens, staff may undertreat pain, which fuels agitation and delirium, reduces sleep, and undermines physiotherapy.

Delirium cascade: delirium increases restraint/PRN antipsychotic use and reduces therapy tolerance; untreated pain and immobility, in turn, perpetuate delirium, a self-reinforcing loop [10,20].

Discharge planning complexity: cognitively impaired patients often require new care packages or placements; delays in assessments and bed availability can prolong stays and increase institutional discharges, independent of medical stability.

Implications for practice and quality improvement

Fast-track theatre for cognitive impairment: treat dementia/delirium as an operational priority flag pre-emptive capacity assessments, early best-interest documentation, and “ready to go” consent plans can protect first-start slots and reduce breaches [3,4,6].

Delirium bundles from admission: universal 4AT on arrival with multicomponent prevention/management (orientation cues, sleep protection, vision/hearing aids, early catheter removal, hydration, mobilisation prompts) reduces incident delirium and severity [10,20].

Dementia-friendly analgesia: standardise scheduled paracetamol/NSAID (where safe), define PRN opioid triggers using PAINAD or similar scales, and include nurse-initiated protocols with mandatory post-dose reassessment. Audit “prescribed vs. administered” gaps as a safety metric [17-19].

Mobilisation first: protect physiotherapy staffing for POD0-2 and use shorter, more frequent sessions with carer participation; couple with analgesia timing to “dose-align” therapy.

Equity-focused metrics: add cognitive-status stratifiers to best-practice dashboards (≤36 h to theatre, POD1/2 mobilisation rates, discharge home proportion, inpatient mortality). Publishing these internally helps sustain focus and accountability.

Discharge acceleration: early referral to discharge-to-assess, rapid care-package broking, and dementia-specialist OT involvement may reduce avoidable institutionalisation [9,10].

Alternative explanations and robustness

Although baseline age and sex were similar by design, residual differences in frailty, ASA grade, comorbidity, or fracture/surgery mix could partially explain outcome differences. Our analysis was unadjusted and descriptive; however, the direction and magnitude of effects are aligned with prior adjusted studies [1-4,9,10,17-20], and the convergence of multiple process gaps (time-to-theatre, analgesia administration, mobilisation) strengthens causal plausibility. Missing timestamp data reduced the sample for some time-based analyses; sensitivity checks restricted to complete cases yielded consistent inferences (data shown in Results).

Generalisability

Findings derive from a single NHS trust with established orthogeriatric co-management and national best-practice targets; external validity should be strongest for similar systems. Nonetheless, the identified bottlenecks, consent workflow, analgesia delivery, and mobilisation under delirium, are common in diverse settings, suggesting broader applicability.

Research directions

Pragmatic trials and stepped-wedge QI studies are warranted to test: (i) fast-track lists prioritising cognitive impairment; (ii) protocolised dementia-friendly analgesia using observational scales; (iii) implementation of multicomponent delirium bundles with fidelity monitoring; and (iv) mobilisation “dose-alignment” with analgesia. Future observational work should adjust for frailty indices, ASA, fracture pattern, and procedure, and incorporate patient-centred outcomes (pain distress metrics, delirium duration, days alive and at home).

Overall interpretation

Within a modern pathway designed for equity, cognitively impaired patients still encounter systematic disadvantages at key perioperative steps that plausibly drive worse outcomes. Focusing improvement efforts on how we consent, when we operate, how we deliver analgesia, and how we mobilise this subgroup offers a realistic route to narrow the gap and improve both survival and independence after hip fracture [1-4,9-10,16-21].

Limitations

This single-centre retrospective evaluation is subject to residual confounding and information bias despite random sampling and data checks. Analyses are primarily unadjusted and should be interpreted as associations rather than causal effects. Delirium was identified from admission 4AT and documentation, so incident/hypoactive delirium may be under-recognised. Analgesia administration was abstracted from the Electronic Prescribing and Medicines Administration (EPMA) system and may be under-recorded. Rehabilitation intensity, ward environment, and social support, potential mediators of discharge destination, were not measured. Missing/incomplete timestamps limited some time-based analyses (complete-case numbers are reported in Results). Missing core timestamps excluded 70/382 records; because missingness was largely operational and similar across groups, any bias from complete-case analysis is uncertain but likely attenuates between-group differences if nondifferential. Precision for key estimates was acceptable, but some subgroup analyses may be underpowered; generalisability is greatest to similar orthogeriatric services.

## Conclusions

In a modern orthogeriatric service, cognitive impairment (dementia and/or delirium) remained a consistent marker of disadvantage across the hip-fracture pathway: patients waited longer for surgery, mobilised later, were less often discharged home, and had higher in-hospital mortality than cognitively intact peers, despite comparable access to geriatric assessment. The convergence of process gaps (time-to-theatre, delivery of analgesia rather than prescribing alone, and mobilisation under delirium) with worse outcomes points to modifiable system factors, not inevitable clinical complexity.

These findings have immediate operational relevance. Equity in hip-fracture care will be achieved not only by meeting headline standards, but also by ensuring those standards are met equitably for people with cognitive impairment. Embedding dementia-informed pathways and monitoring equity-stratified metrics (e.g., time-to-theatre and POD1/2 mobilisation by cognitive status) offers a pragmatic route to narrow the gap within existing resources. While observational and single-centred, the internal consistency of effects across multiple endpoints and their alignment with prior literature support the credibility and generalisability of the signal to similar NHS settings. Prospective evaluation of dementia-informed adaptations is warranted. In the interim, services should treat cognitive impairment as a priority flag across perioperative workflow so that care is safer, timelier, and genuinely equitable for this high-risk population.
